# Complications of percutaneous transhepatic cholangiography and biliary drainage, a multicenter observational study

**DOI:** 10.1007/s00261-021-03207-4

**Published:** 2021-08-06

**Authors:** Ayla S. Turan, Sjoerd Jenniskens, Jasper M. Martens, Matthieu J. C. M. Rutten, Lonneke S. F. Yo, Marco J. L. van Strijen, Joost P. H. Drenth, Peter D. Siersema, Erwin J. M. van Geenen

**Affiliations:** 1 Department of Gastroenterology and Hepatology, Radboud University Medical Center, Nijmegen, The Netherlands; 2 Department of Radiology and Nuclear Medicine, Radboud University Medical Center, Nijmegen, The Netherlands; 3 Department of Radiology, Rijnstate Hospital, Arnhem, The Netherlands; 4 Department of Radiology, Jeroen Bosch Hospital’s, Hertogenbosch, The Netherlands; 5 Department of Radiology, Catharina Hospital, Eindhoven, The Netherlands; 6 Department of Radiology, St Antonius Hospital, Nieuwegein, The Netherlands

**Keywords:** PTC (D), Complications, Infection, Prophylaxis, Antibiotics

## Abstract

**Objectives:**

Over 2500 percutaneous transhepatic cholangiography and biliary drainage (PTCD) procedures are yearly performed in the Netherlands. Most interventions are performed for treatment of biliary obstruction following unsuccessful endoscopic biliary cannulation. Our aim was to evaluate complication rates and risk factors for complications in PTCD patients after failed ERCP.

**Methods:**

We performed an observational study collecting data from a cohort that was subjected to PTCD during a 5-year period in one academic and four teaching hospitals. Primary objective was the development of infectious (sepsis, cholangitis, abscess, or cholecystitis) and non-infectious complications (bile leakage, severe hemorrhage, etc.) and mortality within 30 days of the procedure. Subsequently, risk factors for complications and mortality were analyzed with a multilevel logistic regression analysis.

**Results:**

A total of 331 patients underwent PTCD of whom 205 (61.9%) developed PTCD-related complications. Of the 224 patients without a pre-existent infection, 91 (40.6%) developed infectious complications, i.e., cholangitis in 26.3%, sepsis in 24.6%, abscess formation in 2.7%, and cholecystitis in 1.3%. Non-infectious complications developed in 114 of 331 patients (34.4%). 30-day mortality was 17.2% (*N* = 57). Risk factors for infectious complications included internal drainage and drain obstruction, while multiple re-interventions were a risk factor for non-infectious complications.

**Conclusion:**

Both infectious and non-infectious complications are frequent after PTCD, most often due to biliary drain obstruction.

**Supplementary Information:**

The online version contains supplementary material available at 10.1007/s00261-021-03207-4.

## Introduction

Biliary tract occlusions occur when the biliary system is clogged from within by bile stones or sludge, or when the bile ducts are narrowed by a stricture or due to compression by an external mass. Stasis of bile flow can manifest clinically as jaundice and fatty stools. Furthermore, bacterial colonization in the obstructed biliary system can lead to infections such as cholangitis. Endoscopic Retrograde Cholangiopancreatography (ERCP) is the current gold standard for obtaining biliary access to treat such biliary tract disorders [1, 2]. A drain or stent is placed to restore bile flow from the liver to the gastrointestinal (GI) tract and to keep the common bile duct open. Cannulation rates for ERCP are > 90%, but series have shown failure rates in up to 25%, mostly due to surgically altered anatomy, gastric outlet obstruction, a periampullary diverticulum, an indwelling duodenal stent, or a large tumor [3–7].

In case of cannulation failure of ERCP, in general three alternative strategies are available to access the biliary tree. The first is a repeat ERCP after 3 days by an expert endoscopist, which is successful in 63–78% of cases [8]. Percutaneous transhepatic biliary drainage [PTC (D)] is another procedure for biliary tree drainage resulting in biliary access in 63–86% of cases [1–3]. More recently, endoscopic ultrasound-guided biliary drainage (EUS-BD) was introduced as an alternative to PTCD in selected patient groups.

A German single center study also reported a complication rate of 40% in a cohort of 385 PTCD patients [5]. Furthermore, a high mortality rate was reported, i.e., 23% of patients with a benign disorder died after a median of 192 days after the procedure, while those with malignant obstruction had a mortality rate of 70% after a median of 58 days [5].

These studies have raised concerns about the safety of PTCD. A direct translation of abovementioned data to an average ERCP-failure population is difficult, because the reported studies are derived from mostly small, retrospective, and monocenter studies, performed in subgroups of patients. In addition, risk factors associated with complications and reinterventions as a result of PTCD remain to be mapped. Therefore, we aimed to evaluate safety and associated reintervention rates of PTCD in a real-world population obtained from multiple centers.

## Patients and methods

A total of 331 patients, who underwent PTCD between 2011 and 2016 in 5 centers, one university hospital and four teaching hospitals in the Netherlands, were evaluated. All PTCD were performed after a failed ERCP as a rescue intervention. This study was approved by the medical ethical committees of all participating hospitals (reference number: 2016-0862).

Inclusion criteria were age ≥ 18 years old, one or more successful PTCDs, and at least 1-month follow-up (as registered in the patient’s medical file). Patients with pre-existing infections or fever at the time of the PTCD, or who were currently still receiving antibiotics for an infection in the previous 2 weeks were separately included for assessment of non-infectious complications (for definitions see below).

Primary endpoints were complications and mortality rates within 30 days after PTCD. Complications were divided into non-infectious (bleeding, bile leakage, and stent dislocations) and infectious complications (cholangitis, sepsis, abscess, and cholecystitis).

Secondary endpoints were number of re-interventions during the follow-up time, including anticipated re-interventions such as stent placement, risk factors, and protective factors for complications. The following risk factors were predefined: malignant origin of biliary obstruction, prophylactic antibiotics or not, direct duodenal cannulation, age, BMI > 30, diabetes, bilateral drainage, and whether duodenal cannulation was obtained. After data collection was completed, a random sample of 10% of the data was double checked and uncertainties were resolved through discussion with the involved physicians (EVG, JM, MR, LY, MVS).

### Definitions

In this study, a complication is a drainage-related event within 30 days after the last drainage procedure which requires medical treatment. The definitions that were used for the primary endpoints closely reflect clinical practice (See Supplementary Table 1).

### Statistical analysis

The statistical analysis was performed with IBM SPSS Statistics 25 (IBM Corporation, Armonk, NY, USA). For the primary endpoint, descriptive statistics for patients with infectious and non-infectious complications were calculated. Next, Intra Class Correlations (ICC) were calculated per outcome to determine variance between the five hospitals. Multilevel multivariable logistic regression analysis was performed to determine risk factors or protecting factors for infectious and non-infectious complications. Potential risk factors with baseline differences with *p* ≤ 0.2 from univariate analysis were included in the multilevel model, as well as some pre-defined risk factors based on the literature. Drain malfunction was considered as a potential risk factor for infectious complications in our cohort as most of these preceded infections in the same patients in time. The outcomes of the multivariable models are expressed as odds ratio’s (OR) with 95% confidence intervals (95% CI), with OR = 1 representing no added effect of the variable to affect the outcome. *P*-values < 0.05 were considered statistically significant.

As the variance between patients from different hospitals necessitated a multilevel analysis, we also performed an explanatory subgroup analysis between the five participating centers. Chi^2^ was used for nominal variables (e.g., antibiotic prophylaxis), whereas scale variables (e.g., age, BMI) were analyzed with ANOVA for multiple groups. In case of significant variation, post hoc analyses were performed in Excel using Bonferroni correction with an adjusted *p* < 0.007.

## Results

### General characteristics

In total, 429 patients who were treated with PTCD were evaluated for inclusion. 108 patients were excluded because of loss to follow-up, leaving 331 patients for inclusion with 30-day follow-up after PTCD (see Supplement Fig. 1). Non-infectious complications were analyzed for all patients. We excluded 107 patients for the analysis of infectious complications, because of a pre-existing infection defined as fever, a clinical diagnosis, or suspicion of a systemic infection irrespective of the infection focus, or treatment with antibiotics for such an infection within 7 days prior to PTCD. The indications for PTCD were malignant biliary obstruction or metastasis (194 patients), or benign obstruction (30 patients). Baseline characteristics are shown in Table 1.

**Table 1:** Baseline characteristics of the study groups

Characteristics	Total cohort	Total included in infection analysis
Total	331	224
Gender: Female/Male	160/171 (48.3%/51.7%)	113/111 (50.4%/49.6%)
Age: Mean (SD*)	67 (11.5)	68 (10.9)
Range	29–94	29–94
Age > 70 years	151 (45.6%)	98 (43.8%)
BMI: Mean (SD*)	24.4 (4.17)	24.4 (4.1)
Range	16.4–42.5	16.8–42.5
Immunosuppressant medication	60 (18.1%)	33 (14.7%)
Connection drain duodenum	261 (78.9%)	176 (78.6%)
Mean numbers of re-interventions within 30 days (range)	1 (0–9)	1 (0–9)
Malignant disease	251 (75.8%)	194 (86.6%)
PTC (D) approach		
Left	193	133
Right	91	62
Central	5	4
Both (2 punctures)	20	13
Diabetes Mellitus	85 (25.6%)	52 (23%)
Previous biliary surgery	45 (13.6%)	17 (7.6%)
Prophylactic antibiotics	NA**	127 (56.7%)

*SD standard deviation, **NA Not applicable

### Primary endpoint

The 30-day overall complication rate in our cohort was 62.8%. Non-infectious complications were seen in 34.7% while infectious complications occurred in 40.6%.

Of the 107 patients with an established infection prior to PTCD, 39 (36.4%) developed a non-infectious complication, mainly drain obstruction due to sludge or dislocation. This was similar to the 33.5% non-infectious complication rate in the group of 224 patients, without pre-existing infectious complications (*p* = 0.71). A substantial overlap in non-infectious and infectious complications was observed and 55 patients developed both types of complications.

The overall 30-day all-cause mortality rate was 17.2% (*N* = 57) (see Table 2). At least 30 deaths were directly related to the underlying malignancy and were not PTCD related. No periprocedural mortality occurred during PTCD. Nonetheless, a total of 27 deaths (8.2%) seemed to be a direct or indirect result of the PTCD procedure, as they occurred while being treated for a complication, such as bacteremia or sepsis.

**Table 2:** Complications after PTCD

Outcome	Total cohort	Pre-existing infections	No pre-existing infections
Cumulative complication rate	61.9% (205/331)	36.4% (39/107)	58.9% (132/224)
Infectious complications	–	–	40.6% (91/224)
Cholangitis	–	–	26.3% (59/224)
Sepsis	–	–	24.6% (55/224)
Abscess	–	–	2.7% (6/224)
Cholecystitis	–	–	1.3% (3/224)
Non-infectious complications	34.4% (114/331)	36.4% (39/107)	33.5% (75/224)
Severe hemorrhage	6.9% (23/331)	7.5% (8/107)	6.7% (15/224)
Peritonitis	2.7% (9/331)	3.7% (4/107)	2.2% (5/224)
Bile leakage/biloma	28.7% (95/331)	30.8% (33/107)	27.7% (62/224)
All-cause mortality	17.2% (57/331)	19.6% (21/107)	16.1% (36/224)

### Secondary endpoints: risk factor analysis

In 224 patients, a re-intervention was performed within 30 days after the initial PTCD, resulting in a re-intervention rate of 73.7%. In 131 patients initially a drain was placed, which was replaced in 67 patients by an internalized stent within days to weeks. In 26 patients no drain was placed, because a stent was already placed during the initial PTCD. No difference in obstruction rates was found between stents and drains (46.3% vs 37.8% respectively, *p* = 0.295).

Three multilevel logistic regression models with backward elimination were run to elicit risk factors for the outcomes non-infectious complications (ICC = 0.01) and infectious complications (ICC = 0.07). See Supplement Table 3 for additional post hoc analysis of intra-hospital variation.

Risk factors for non-infectious complications were analyzed in the total group of 331 patients. The univariate analysis for non-infectious complications resulted in three variables with a *p* < 0.20 that were included in the multivariate model, i.e., BMI, mean number of re-interventions within 30 days and antibiotic prophylaxis (see Table 3). The number of re-interventions within 30-day follow-up was the only significant risk factor for non-infectious complications (OR 1.52, 95% CI 1.27–1.83, *p* = 0.00).

**Table 3:** Risk factors for non-infectious complications

	Univariate OR (95% CI)	*P*-value	Multivariate OR (95% CI)	*P*-value
Female gender	0.99 (0.37–2.65)	0.98		
Age	1.02 (0.97–1.07)	0.46		
BMI	1.10 (0.98–1.22)	0.10	0.99 (0.94–1.04)	0.65
Internalized drainage	2.32 (0.50–10.80)	0.28		
Re-interventions within 30 days	1.52 (1.27–1.82)	0.00	1.52 (1.27–1.83)	0.00
Malignant disease	0.81 (0.51–1.28)	0.36		
Diabetes Mellitus	0.80 (0.44–1.44)	0.45		
Smoker	0.75 (0.27–2.06)	0.58		
Medical history of biliary surgery	1.26 (0.66–2.42)	0.48		
Prophylactic Antibiotics	0.53 (0.20–1.42)	0.20		

Infectious complications were seen in the 224 patients who were not known with a prior infection. The univariate analysis in this group resulted in five variables with a *p* < 0.20 that were included in the multivariate model, i.e., position of the drain in the GI tract, mean number of procedures within 30 days, catheter obstruction, diabetes mellitus, and prophylactic antibiotics (see Table 4). Only position of the drain in the GI tract and catheter obstruction remained statistically significant in the multilevel logistic regression model with an OR of 2.12 (95% CI 1.03–4.38, *p* = 0.042) and 2.60 (95% CI 1.39–4.88, *p* = 0.003), respectively.

**Table 4:** Risk factors for infectious complications

	Univariate OR (95% CI)	*P*-value	Multivariate OR (95% CI)	*P*-value	Multivariate after backward elimination OR (95% CI)	*P*-value
Female gender	0.80 (0.46–1.38)	0.42				
Age	1.00 (0.98–1.03)	0.79				
BMI	1.05 (0.98–1.12)	< 0.20	1.06 (0.99–1.14)	0.11		
Immunosuppressant medication	1.10 (0.50–2.40)	0.81				
Internalized drainage	2.14 (1.05–4.36)	0.04	2.08 (0.99–4.38)	0.05	2.12 (1.03–3.38)	0.04
Re-interventions within 30 days	1.29 (1.03–1.62)	0.03	1.18 (0.92–1.50)	0.20		
Malignant disease	1.20 (0.52–2.78)	0.67				
Catheter obstruction	2.61 (1.40–4.87)	< 0.00	2.24 (1.12–4.49)	0.02	2.60 (1.39–4.88)	< 0.00
Diabetes Mellitus	0.64 (0.31–1.33)	0.23				
Smoking	0.79 (0.46–1.38)	0.41				
Previous biliary surgery	1.26 (0.06–25.89)	0.87				
Prophylactic Antibiotics	0.80 (0.44–1.44)	0.45				

Of the 54 patients with clinical sepsis, 48 patients had blood samples taken for culture of which 42 had a positive blood culture. In the 12 clinical sepsis cases without a positive blood culture, antibiotic treatment was started immediately after the procedure or after the presentation of symptoms. Reasons to start early antibiotic treatment were mainly a difficult procedure or an increase in symptoms such as pain or (sub-) febrile temperature. *Escherichia coli* was cultured from blood cultures of twelve patients, *Enterobacter cloacae* from six, and *Enterococcus faecium* from five. Other common bacteria included *Klebsiella pneumoniae* (three), *Klebsiella oxytoca* (three), Streptococcae (three), and *Pseudomonas aeruginosa* (two). The most commonly prescribed antibiotic in these cases was piperacillin/tazobactam.

## Discussion

The overall incidence of complications and mortality after PTCD is substantial. More than 50% of patients in our cohort developed one or more drainage-related complications after PTCD, mainly cholangitis and sepsis. Mortality seemed to be a direct or indirect result of the PTCD procedure in 27 patients. It is important to keep in mind that all PTCD procedures were performed after a failed ERCP as a rescue intervention. Therefore, these complications may also be the consequence of the previously performed ERCP which may have played a role as well in the significant 30-day morbidity and mortality rates. The majority of these patients had malignant biliary obstruction, further increasing the baseline risk of infectious complications after PTCD. Nonetheless, our study is not the first study that reports complication rates after PTCD that are higher than the threshold as stated in recent SIR guidelines [9]. In 2016, another Dutch RCT comparing the efficacy and safety of pre-operative drainage by ERCP versus PTCD in perihilar cholangiocarcinoma was terminated prematurely because of increased overall mortality rates in the PTCD group (3/27 (11%) vs. 11/27 (41%), respectively) [7]. Cholangitis occurred in 59% in the PTCD-group vs. 37% in the ERCP-group. These findings are in line with our results and clearly shows that infectious complications are common in patients with malignant biliary obstruction, are often drainage-related and seem to occur more often after PTCD than ERCP.

The all-cause mortality rate in our cohort was comparable to earlier studies with 30-day mortality rates of 10–23.1% [6, 10–12]. We found a high proportion (40.6%) of PTCD-related infectious complications. Previous studies have reported lower infectious complication rates of up to 17% [5,6, 12–17]. A possible explanation for these substantial morbidity rates could be the clinical definition of our endpoints. For example, the clinical diagnosis of sepsis following PTCD did not depend on a confirmative blood culture, because in clinical practice blood cultures were often not drawn or only obtained after the start of antibiotic therapy. Another explanation could be the high proportion of patients with a malignant biliary obstruction in our study, a known risk factor for cholangitis and sepsis [11, 18]. Patients with a malignancy usually have a poorer performance status at baseline compared to patients with a benign obstruction [15]. Nonetheless, we did not identify malignant disease as a risk factor for infectious complications. The number of observations of patients with benign obstructions in our study may well have been too low for a robust comparison between these groups.

After the procedure, the majority of patients (78.6%) in our cohort had internalized drainage. This is desirable as it decreases the risk of metabolic disturbances and likely increases patients’ quality of life [19]. Nonetheless, our results show that internalized drainage was an independent risk factor for infectious complications. This seemed to be related to an increase in catheter obstructions, resulting in biliary stasis and cholangitis. These findings are in line with a study that compared complication rates between internal–external drains with a capped-off external component, and external drainage. The authors reasoned that translocation of intestinal flora may have caused (infectious) complications, due to a retrograde flow of intestinal content through the internalized drain [20].

Severity grading is a tool that can potentially discriminate between non severe, moderate severe, and severe complications, and is of utmost importance for future research and quality control in PTCD. Several scores can be used to classify the severity of PTCD complications, for example the ASGE ERCP severity grading and the Clavien-Dindo post-operative complication severity grading [21]. Unfortunately, these scores could not be calculated, because of the incomplete data present in the electronic medical record.

In our study, antibiotic prophylaxis did not prevent infectious complications after PTCD in patients with malignant obstruction. This could possibly be explained by varying antibiotic prophylaxis practices in the Netherlands; we noted a wide variation in timing, type, dosing, and duration of antibiotic prophylaxis. According to the literature, antibiotic prophylaxis prior to PTCD decreases cholangitis risk from 24–46% to 4.6%. [15, 22] Therefore, the 2010 Cardiovascular Interventional Radiological Society of Europe and the Canadian Interventional Radiology Association guideline, advocate antibiotic prophylaxis in PTCD procedures based on level 3–5 evidence [3, 22, 23]. Notwithstanding this, there is currently no standard protocol for antibiotic prophylaxis in PTCD in the Netherlands. Based on the high rate of infectious complications in our study and guideline recommendations, we therefore suggest prescribing pre-interventional intravenous antibiotics to all patients.

This study comes with strengths and limitations. The multicenter design is a major strength of our study. In this large heterogeneous real-life cohort, we report both infectious and non-infectious complications and their associations. Due to the pragmatic definition of endpoints, adjusted to the existing clinical documentation, the results closely mirror everyday clinical practice. Additionally, risk factors for complications and for PTCD-related mortality were analyzed. A strength in our analysis is that we considered the trend of drain obstruction preceding infectious complications in time. Therefore, we were able to confirm that drain obstruction due to sludge or dislocation is an independent risk factor for infectious complications. Furthermore, the multilevel analysis takes the multicenter character of our cohort into account, correcting the OR for any differences between patients from different sites.

Nevertheless, we recognize that the retrospective design of this study has its limitations. The study design is more prone to bias as subjects were not randomized and data collection was dependent on availability and accuracy of registration and follow-up information. Antibiotic prophylaxis strategies differ between hospitals and detailed data such as time of drug administration were often not retrievable. Also, the exact time relation between PTCD and subsequent complications was not structurally documented in the case record form. These factors limit the distinction between early and late complications and additional analysis of the effect of antibiotic prophylaxis in these two groups (See Supplementary Table 2 and Fig. 2). Future studies should focus on clinically relevant definitions of complications, severity grading of complications, quality of life, and comparison of PTCD with advanced endoscopic techniques, such as EUS-guided biliary drainage. Recently, a meta-analysis reported a significant superior performance of EUS-guided biliary drainage compared to PTCD in patients with a malignant obstruction in the common bile duct in terms of technical success (92% vs. 86%, respectively), complication rates (16% vs 80%, respectively), and reintervention rates (16% vs 45%, respectively) [4].

In conclusion, this study shows that PTCD following unsuccessful ERCP is associated with a high number of adverse events, in particular cholangitis and sepsis. The high complication rates justify quality control of the PTCD procedure and pre-procedural prophylactic antibiotic treatment.

## Supplementary Information

Below is the link to the electronic supplementary material.

Supplementary Figure 1. Flowchart of inclusion. Of the 224 patients, eight patients had a history of pylorus preserving pancreaticoduodenectomy (PPPD) or Whipple surgery. Ten patients received Whipple or PPPD within the 30 days follow-up. As the operations were in the final week of follow up, consensus was to not exclude these cases from the analyses. Potential complications after surgery were not counted as PTC(D)-related complications. Supplementary file1 (JPG 21 KB)

Supplementary Figure 2. Effect of antibiotic prophylaxis on infectious complications after PTCD. A difference was seen in complication rates between 127 patients with and 97 patients without antibiotic prophylaxis. However, this was not statistically significant. Supplementary file2 (JPG 905 KB)

Supplementary file3 (DOCX 18 KB)

Supplementary file4 (DOCX 15 KB)

Supplementary file5 (DOCX 16 KB)

## Abbreviations

Abp: Antibiotic Prophylaxis
ASGE: American Society for Gastrointestinal Endoscopy
BMI: Body Mass Index
95% CI: 95% Confidence Interval
ERCP: Endoscopic Retrograde Cholangiopancreatography
EUS-BD: Endoscopic Ultrasound-Guided Biliary Drainage
GI: Gastrointestinal
ICC: Intra Class Correlations
NA: Not applicable
OR: Odds Ratio
PTCD: Percutaneous Transhepatic Biliary Drainage
SD: Standard Deviation
