# Supplementary figures and images for: Complications of percutaneous transhepatic cholangiography and biliary drainage, a multicenter observational study
Source: Abdom Radiol (NY). 2021 Aug 6;47(9):3338–44. doi: 10.1007/s00261-021-03207-4 (PMC9388415; doi:10.1007/s00261-021-03207-4)

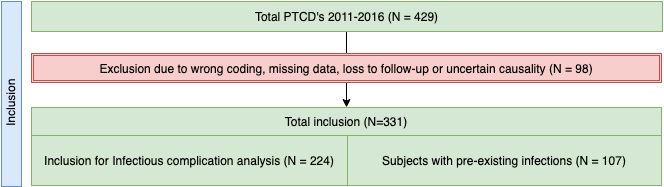

Supplement: Supplementary file 1 — Supplementary Figure 1. Flowchart of inclusion. Of the 224 patients, eight patients had a history of pylorus preserving pancreaticoduodenectomy (PPPD) or Whipple surgery. Ten patients received Whipple or PPPD within the 30 days follow-up. As the operations were in the final week of follow up, consensus was to not exclude these cases from the analyses. Potential complications after surgery were not counted as PTC(D)-related complications. Supplementary file1 (JPG 21 KB) [file 261_2021_3207_MOESM1_ESM.jpg]

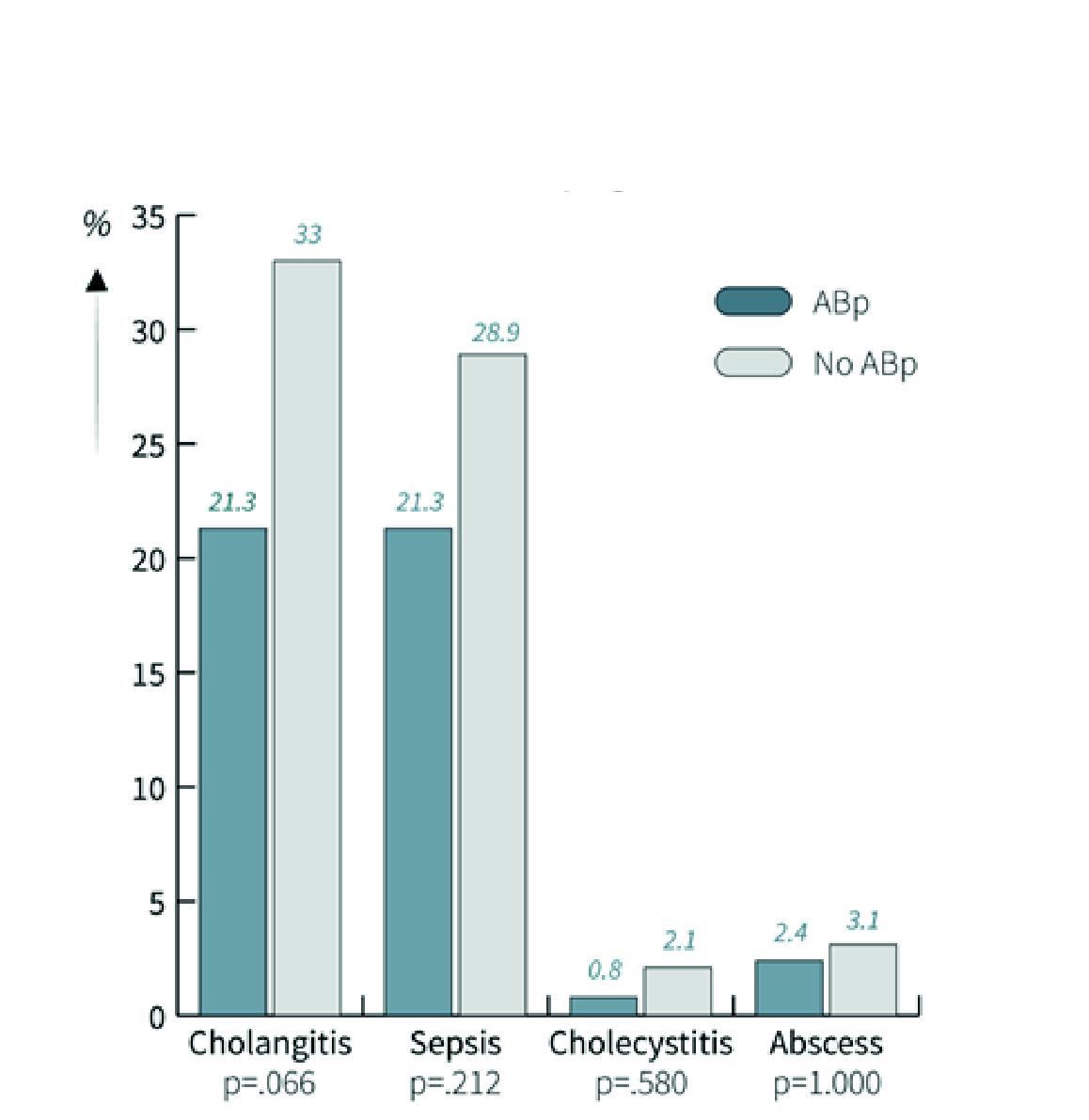

Supplement: Supplementary file 2 — Supplementary Figure 2. Effect of antibiotic prophylaxis on infectious complications after PTCD. A difference was seen in complication rates between 127 patients with and 97 patients without antibiotic prophylaxis. However, this was not statistically significant. Supplementary file2 (JPG 905 KB) [file 261_2021_3207_MOESM2_ESM.jpg]
