# Supplementary material for: Complications of percutaneous transhepatic cholangiography and biliary drainage, a multicenter observational study
Source: Abdom Radiol (NY). 2021 Aug 6;47(9):3338–44. doi: 10.1007/s00261-021-03207-4 (PMC9388415; doi:10.1007/s00261-021-03207-4)
Supplement: Supplementary file 3 — Supplementary file3 (DOCX 18 KB) [file 261_2021_3207_MOESM3_ESM.docx]

**Supplementary Table 1. Definitions of complications**

| **Non-infectious complications** | Complications caused by manipulation of the biliary tree during the PTC(D) procedure, e.g. the puncturing of the central bile duct. |
| --- | --- |
| Leakage | Outflow of bile from the exit wound of the PTC(D) alongside the drain and included both outward leakage and internal leakage such as biloma on imaging. |
| Bleeding | Outflow of blood through the drain and/or internal bleeding necessitating additional therapeutic interventions such as blood transfusion or re-PTC(D). |
| **Infectious complications** | Complications caused by bacterial translocation, e.g. intestinal flora entering the bile duct or bacteria entering the circulation through the PTC(D) entry in the biliary system. |
| Cholangitis | Clinical diagnosis of infected bile ducts documented in the patient record. Compliance with the Tokyo guidelines was assumed, but not checked. |
| Cholecystitis | Diagnosis of infection of the gallbladder wall as documented in the patient record, according to the Tokyo guidelines 2018.^23^ Variables leading to this diagnosis were not individually checked. |
| Sepsis | Intravasation of microorganisms into the bloodstream due to mechanical agitation of an infected biliary system, presenting clinically with fever and/or cold shakes, tachycardia and/or hypotension and preferably confirmed with a positive blood culture. This also includes cases without a positive blood culture, but where nevertheless the local sepsis protocol was initiated and antibiotics were started. |
| Abscess | Localized collections of necrotic inflammatory tissue caused by bacterial, parasitic or fungal agents in the right upper abdomen, developed after PTC(D), diagnosed on CT imaging. |
