# Supplementary material for: Complications of percutaneous transhepatic cholangiography and biliary drainage, a multicenter observational study
Source: Abdom Radiol (NY). 2021 Aug 6;47(9):3338–44. doi: 10.1007/s00261-021-03207-4 (PMC9388415; doi:10.1007/s00261-021-03207-4)
Supplement: Supplementary file 4 — Supplementary file4 (DOCX 15 KB) [file 261_2021_3207_MOESM4_ESM.docx]

**Supplementary Table 2. Analysis of antibiotic prophylaxis and complications**

| **Outcome** | ***Antibiotic prophylaxis group %, (n/N)*** | ***No Antibiotic prophylaxis group %, (n/N)*** | ***P-value*** |
| --- | --- | --- | --- |
| Cumulative complication rate  (N with any complication) | 55.1%, (70/127) | 63.9%, (62/97) | 0.22 |
| Infectious complications | 35.4%, (45/127) | 47.4%, (46/97) | 0.08 |
| - Cholangitis | 21.3%, (27/127) | 33%, (32/97) | 0.07 |
| - Sepsis | 21.3%, (27/127) | 28.9%, (28/97) | 0.21 |
| - Abscess | 2.4%, (3/127) | 3.1%, (3/97) | 1.00 |
| - Cholecystitis | 0.8%, (1/127) | 2.1%, (2/97) | 0.58 |
| Non-infectious complications | 33.9%, (43/127) | 33.0%, (32/97) | 1.00 |
| - Severe Hemorrhage | 7.9%, (10/127) | 5.2%, (5/97) | 0.59 |
| - Peritonitis | 2.4%, (3/127) | 2.1%, (2/97) | 1.00 |
| - Bile leakage/ biloma | 27.6%, (35/127) | 27.8%, (27/97) | 1.00 |
| All-cause mortality) | 14.2%, (18/127) | 18.6%, (18/97) | 0.46 |
