# Supplementary material for: Complications of percutaneous transhepatic cholangiography and biliary drainage, a multicenter observational study
Source: Abdom Radiol (NY). 2021 Aug 6;47(9):3338–44. doi: 10.1007/s00261-021-03207-4 (PMC9388415; doi:10.1007/s00261-021-03207-4)
Supplement: Supplementary file 5 — Supplementary file5 (DOCX 16 KB) [file 261_2021_3207_MOESM5_ESM.docx]

**Supplementary Table 3. Variability testing between the hospitals.**

|  | **Center 1** | **Center 2** | **Center 3** | **Center 4** | **Center 5** | **Significant variation** |
| --- | --- | --- | --- | --- | --- | --- |
| **Characteristics:** | | | | | | |
| N included | 38 | 64 | 42 | 44 | 36 | - |
| Gender: Female  Male | 17  21 | 29  35 | 25  17 | 23  21 | 19  17 | No (p=0.611) |
| Age: Mean  Spread | 69  46-87 | 65  29-87 | 70  50-94 | 69  31-90 | 67  40-87 | Yes p=0.042* |
| BMI: Mean  Spread | 24.9  16.8-34.4 | 24,4  16.9-42.5 | 23.8  18.1-34.6 | 25.3  17.1-37.5 | 23.6  17.8-32.5 | No (P=0.312) |
| ABp | 84.2% (32/38) | 42.2% (27/64) | 92.9%  (39/42) | 25%  (11/44) | 50%  (18/36) | Yes  p=0.000 *² |
| Mean nr of procedures within 30 days | 1  (0-4) | 2  (0-9) | 1  (0-4) | 1  (0-4) | 1  (0-3) | Yes  p=0.014 |
| Connection to duodenum | 84.2% (32/38) | 73.4% (47/64) | 85.7% (36/42) | 75% (33/44) | 77.8% (28/36) | No (p=0.503) |
| **Outcome:** | | | | | | |
| Infectious complications | 21.1% (8/38) | 45.3% (29/64) | 31%  (13/42) | 56.8%  (25/44) | 44.4%  (16/36) | Yes p=0.010*³ |
| - Cholangitis | 13.2%  (5/38) | 29.7%  (19/64) | 23.8%  (10/42) | 34.1%  (15/44) | 28.8%  (10/36) | No (p=0.257) |
| - Sepsis | 7.9%  (3/38) | 28.1%  (18/64) | 21.4%  (9/42) | 34.1%  (15/44) | 28.8%  (10/36) | No (p=0.069) |
| - Abscess | 2.6%  (1/38) | 1.6%  (1/64) | 2.4%  (1/42) | 2.3%  (1/44) | 5.6%  (2/36) | No (p=0.828) |
| - Cholecystitis | 0 | 3.1%  (2/64) | 0 | 2.3%  (1/44) | 0 | No (p=0.492) |

** Post hoc analyses (LSD) show that patients in Center 2 have a significantly younger age than in Centers 1,3 and 4.*

**² Post hoc analysis with a Bonferroni correction shows that Centers 1 and 3 give significantly more patients ABp prior to PTCD, whereas Center 4 significantly less often provides ABp to PTCD patients.*

*³ *The chi-square test produces p=0.010; post hoc analysis with a Bonferroni correction shows that the difference in infectious complications between the centers is borderline significant for Center 1 with P=0,007. (adjusted α=0.005, p=0.007).*
